# Double-C hold for bag-mask ventilation during resuscitation

**DOI:** 10.1007/s00540-021-02935-7

**Published:** 2021-04-24

**Authors:** Markus Isser, Hannah Salchner, Wolfgang Lederer

**Affiliations:** 1 Medical Division, Mountain Rescue Tyrol, Florianistr. 2, 6410 Telfs, Austria; 2 Department of Anesthesiology and Critical Care Medicine, Medical University of Innsbruck, Anichstr. 35, 6020 Innsbruck, Austria

**Keywords:** Airway control, Basic cardiac life support, Resuscitation

To the Editor:

“Aerosol extractor for airway management of COVID-19 patients” by Tomoyuki Saito, Asuka Fujishiro and Takashi Asai deals with an important topic [1]. Continuous oro-tracheal suctioning can minimize exhaled air dispersion during coughing bouts [2]. We liked to highlight two additional protective measures, in particular, the double-C hold in bag-mask ventilation [3] and blanketing the patient with a thin plastic barrier [4]. A good seal for bag-mask ventilation increases efficacy and reduces the risk of contamination with aerosols.

Blanketing the patient with a thin plastic barrier can diminish the risk of contamination from aerosols, vomitus, blood, and body secretions [4]. The foil conforms to the face with a central perforation to ensure air-tight installation of the mask below the shield with filter, valve and bag above. Aerosols from air leakage are removed by adhesion and drainage below the barrier.

## References

[1] Saito T, Fujishiro A, Asai T (2021). Aerosol extractor for airway management of COVID-19 patients. J Anesth.

[2] Chan MT, Chow BK, Lo T, Ko FW, Ng SS, Gin T, Hui DS (2018). Exhaled air dispersion during bag-mask ventilation and sputum suctioning—implications for infection control. Sci Rep.

[3] Nolan JP, Monsieurs KG, Bossaert L, Böttiger BW, Greif R, Lott C, Madar J, Olasveengen TM, Roehr CC, Semeraro F, Soar J, Van de Voorde P, Zideman DA, Perkins GD; European Resuscitation Council COVID-Guideline Writing Groups (2020). European resuscitation council COVID-19 guidelines executive summary. Resuscitation.

[4] Matava CT, Yu J, Denning S (2020). Clear plastic drapes may be effective at limiting aerosolization and droplet spray during extubation: implications for COVID-19. Can J Anaesth.

